# Retrospective audit of access to care for children and young people diagnosed with localised scleroderma or juvenile systemic sclerosis in the United Kingdom

**DOI:** 10.1186/1546-0096-10-S1-A67

**Published:** 2012-07-13

**Authors:** Daniel P Hawley, Eileen M Baildam, Tania S Amin, Mary K Cruikshank, Joyce E Davidson, Jennifer Dixon, Neil S Martin, Victoria Ohlsson, Clarissa A Pilkington, Satyapal Rangaraj, Philip Riley, Chitra Sundaramoorthy, Jo Walsh, Helen E Foster

**Affiliations:** 1 Derriford Hospital, Plymouth, Devon, UK; 2 Great Ormond Street Hospital, London, UK; 3 Newcastle Hospital's NHS Trust, Newcastle-upon-Tyne, Tyneside, UK; 4 Newcastle Hospitals NHS Trust, Newcastle-upon-Tyne, Tyneside, UK; 5 Nottingham Children's Hospital, Nottingham, Nottinghamshire, UK; 6 Royal Hospital for Sick Children Edinburgh, Edinburgh, Lothian, UK; 7 Royal Hospital for Sick Children Glasgow, Glasgow, Lanarkshire, UK; 8 Royal Hospital for Sick Children Glasgow and Edinburgh, Edinburgh, Lothian, UK; 9 Royal Liverpool Children's Hospital, Liverpool, Mersyside, UK; 10 Royal Manchester Children's Hospital, Manchester, Lancashire, UK

## Purpose

Localised scleroderma (LS) and juvenile systemic sclerosis (jSSc) are very rare paediatric diseases managed by paediatric rheumatologists and dermatologists[1]. Optimal treatment is controversial in the absence of clinical trials, but most paediatric rheumatologists advocate systemic immunosuppression to avoid progressive deformity, functional disability and disfigurement[2]. We aimed to describe pathways through which children and young people receive a diagnosis of LS or jSSc, and to document these pathways from the time the first symptom was noticed by the patient or their family, through to the time when a diagnosis of LS or jSSc was first considered. We also aimed to document the health care professionals involved in these pathways to diagnosis.

## Methods

We performed a retrospective case note audit of existing patients under paediatric rheumatology care who have presented between Jan 2005 – Jan 2010. Data was collected using a piloted proforma. Each participating centre identified children and young people who presented to their services during Jan 2005 – Jan 2010. All data collected was pseudo-anonymised and collected in accordance with Caldicott guidelines. As this was an audit of access to care no ethical approval was required. No patient or family contact was necessary at any time during this project.

## Results

Eight centres provided data on 89 cases: 62 female, 26 male; 73 LS, 16 jSSc. Symptoms were first noticed at a mean age of 6.8 years (*1month–15 years*) for LS and 8.5 years (*9 months – 15 years*) for jSSc. Mean total time from first symptom to seeing a paediatric rheumatologist was 24.2 (*1-103*) months (LS) and 13.7 (*0-50*) months (jSSc). Mean time from first symptom to seeing first health care professional (HCP) was 8.0 (*0-72*) months (LS) and 8.8 (*0-50*) months (jSSc). Mean time from seeing first HCP to seeing a paediatric rheumatologist was 15.5 (*0-103*) months (LS) and 2.9 (*0-10*) months (jSSc). First health care professional (HCP) seen was usually (74%) a General Practitioner (GP). Referring HCP to paediatric rheumatology was usually a dermatologist (56%) for LS, and varied for jSSc. Mean time from first symptom to diagnosis was 21.3 (*1-191*) months (LS) and 15.9 (*1-50*) months (jSSc). The median number of HCPs seen prior to referral to a paediatric rheumatologist was 2 (*1-5*).

## Conclusion

There is a prolonged interval from first symptom to assessment by a paediatric rheumatologist and diagnosis. GPs are usually the initial HCP to assess; dermatologists usually the HCP referring to paediatric rheumatology services. The prolonged interval to diagnosis may adversely affect outcome and there is need to raise awareness of this rare diagnosis and facilitate earlier recognition.

## Disclosure

Daniel P. Hawley: None; Eileen M. Baildam: None; Tania S. Amin: None; Mary K. Cruikshank: None; Joyce E. Davidson: None; Jennifer Dixon: None; Neil S. Martin: None; Victoria Ohlsson: None; Clarissa A. Pilkington: None; Satyapal Rangaraj: None; Philip Riley: None; Chitra Sundaramoorthy: None; Jo Walsh: None; Helen E. Foster: None.

## References

[B1] Herrick AL Ennis H Bhushan M Silman AJ Baildam EM Incidence of childhood linear scleroderma and systemic sclerosis in the UK and Ireland Arthritis Care Res (Hoboken) 2010 62 2 213 8 10.1002/acr.20070 20191520

[B2] Li SC Feldman BM Higgins GC Treatment of pediatric localized scleroderma: results of a survey of North American pediatric rheumatologists J Rheumatol 2010 37 1 175 81 Epub 2009 Nov 16 10.3899/jrheum.090708 19918041

